# Supplementary material for: Structures of ZYG11B-EloB-EloC-substrate complex reveal mechanisms of CRL2ZYG11B assembly and function
Source: Nat Commun. 2026 Mar 31;17:4648. doi: 10.1038/s41467-026-71318-x (PMC13201589; doi:10.1038/s41467-026-71318-x)
Supplement: Supplementary file 1 — Supplementary Information [file 41467_2026_71318_MOESM1_ESM.pdf]

Supplementary file

Structures of ZYG11B-EloB-EloC-substrate complex reveal mechanisms of $CRL2^{ZYG11B}$ assembly and function

Ni Lin[1,2,4], Han Feng[2,4], Yushan Geng[2,3], Yina Gao[2], Miao Shi[1,2], Songqing Liu[2], Pu Gao[2,3,] *, Yong Wang[2,] *

[1] Science and Technology Innovation Center, Shandong First Medical University & Shandong Academy of Medical Sciences, Jinan 250000, China

[2] National Laboratory of Biomacromolecules, CAS Center for Excellence in Biomacromolecules, Institute of Biophysics, Chinese Academy of Sciences, Beijing 100101, China

[3] University of Chinese Academy of Sciences, Beijing 100049, China

[4] These authors contributed equally

* Correspondence: gaopu@ibp.ac.cn (P.G.); wangyong@ibp.ac.cn (Y.W.)

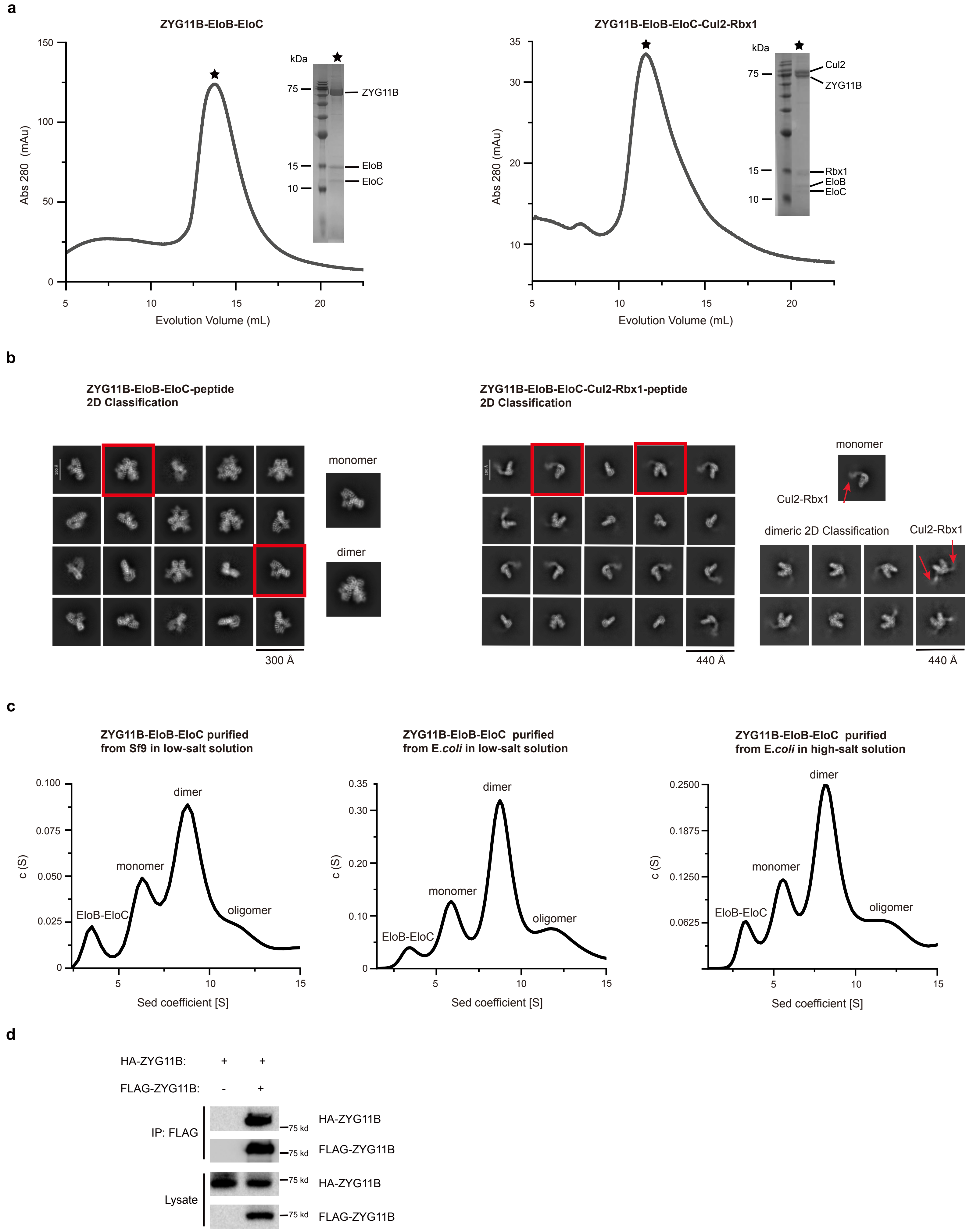

a
ZYG11B-EloB-EloC
Abs 280 (mAu)
Evolution Volume (mL)
kDa
75
15
10
ZYG11B
EloB
EloC
ZYG11B-EloB-EloC-Cul2-Rbx1
Cul2
ZYG11B
Rbx1
b
ZYG11B-EloB-EloC-peptide
2D Classification
monomer
dimer
300 Å
ZYG11B-EloB-EloC-Cul2-Rbx1-peptide
2D Classification
monomer
Cul2-Rbx1
dimeric 2D Classification
440 Å
c
ZYG11B-EloB-EloC purified
from Sf9 in low-salt solution
ZYG11B-EloB-EloC purified
from E.coli in low-salt solution
ZYG11B-EloB-EloC purified
from E.coli in high-salt solution
c (S)
Sed coefficient [S]
EloB-EloC
monomer
dimer
oligomer
d
HA-ZYG11B: + +
FLAG-ZYG11B: - +
IP: FLAG
Lysate
75 kd
HA-ZYG11B
FLAG-ZYG11B

Supplementary Figure 1. Purification and characterization of ZYG11B-EloB-EloC or ZYG11B-EloB-EloC-Cul2-Rbx1. **a,** Size-exclusion chromatography and SDS-PAGE of ZYG11B-EloB-EloC (left) and ZYG11B-EloB-EloC-Cul2-Rbx1 (right) complex on a Superdex 200 Increase 10/300 GL column. Source data are provided as a Source Data file. **b,** Cryo-EM 2D class averages of ZYG11B-EloB-EloC-substrate (left) and ZYG11B-EloB-EloC-Cul2-Rbx1-substrate complex (right) indicate the presence of both monomeric and dimeric state. The Cul2-Rbx1 complex is marked by red arrows. **c,** Analytical ultracent rifugation analysis of ZYG11B-EloB-EloC complex derived from different resources indicate that both monomer and dimer coexist in solution: from Sf9 insect cell in 50 mM NaCl (left), from E. coli in 50 mM NaCl (middle), and from E. coli in 300 mM NaCl (right). **d,** Coimmunoprecipitation analysis shows Flag-ZYG11B interacts with HA-ZYG11B in HEK293T cells, indicat ing that ZYG11B can form a homodimer. Source data are provided as a Source Data file.

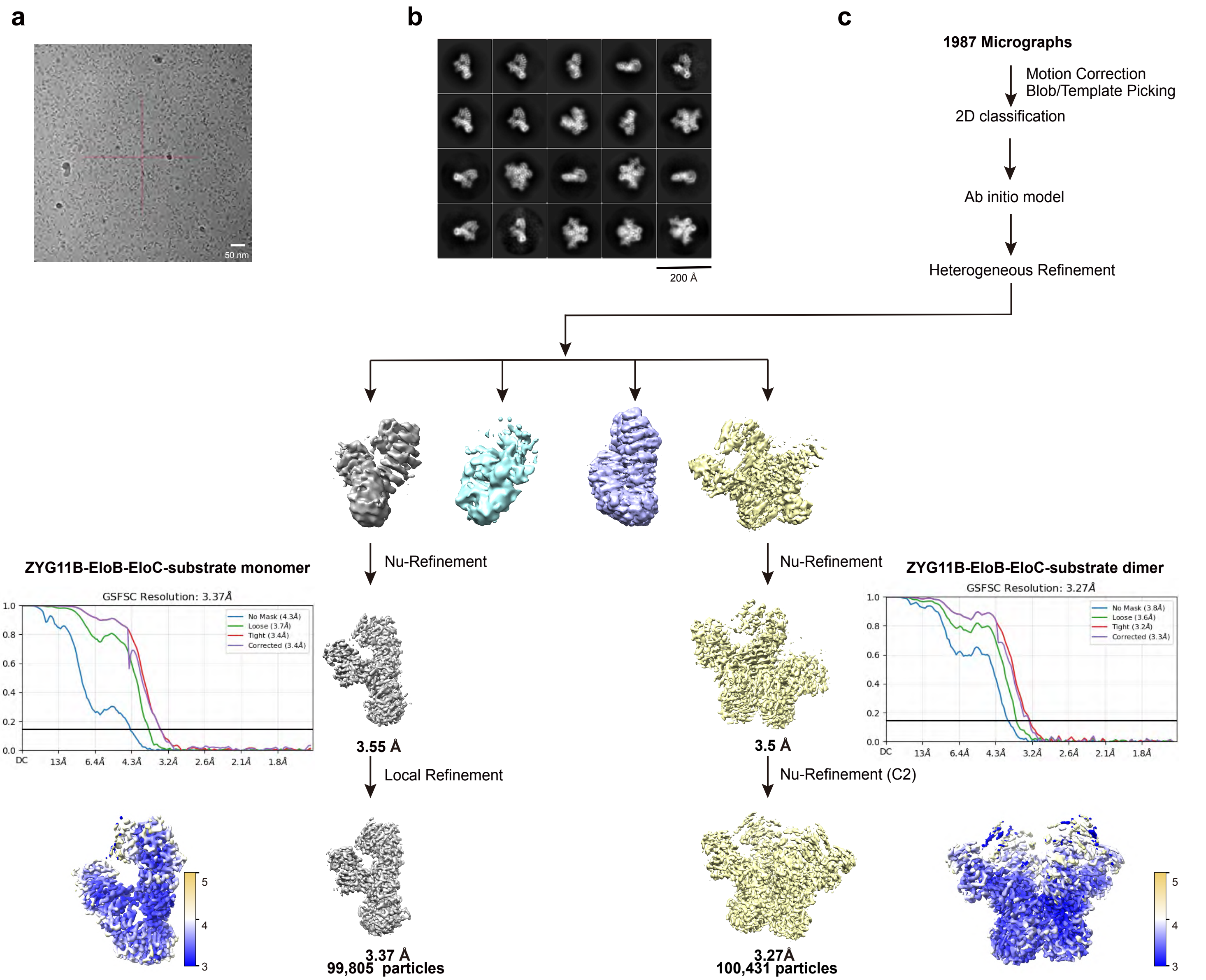

Supplementary Figure 2. Cryo-EM analysis of the ZYG11B-EloB-EloC-substrate complex. a, Representative cryo-EM micrograph of the ZYG11B-EloB-EloC-substrate complex. Scale bars: 50 nm. **b,** Representative 2D class averages of ZYG11B-EloB-EloC-substrate complex. **c,** Cryo-EM data processing workflow, local-resolution maps and FSC curves for the monomeric and dimeric ZYG11B-EloB-EloC-substrate complex.

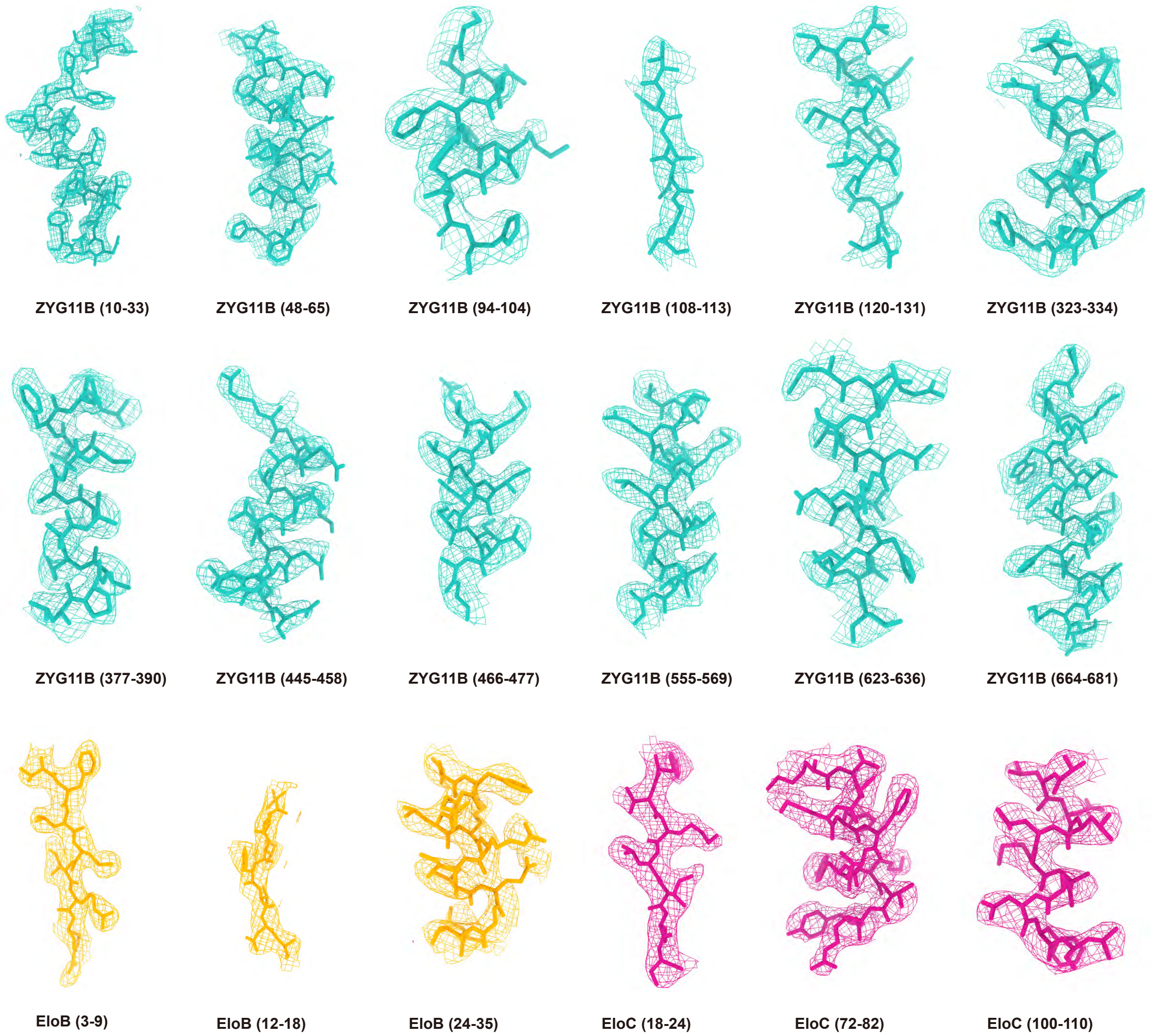

Supplementary Figure 3. Cryo-EM density maps of ZYG11B-EloB-EloC complex. Cryo-EM density maps for various regions of ZYG11B (cyan), EloB (orange) and EloC (magenta).

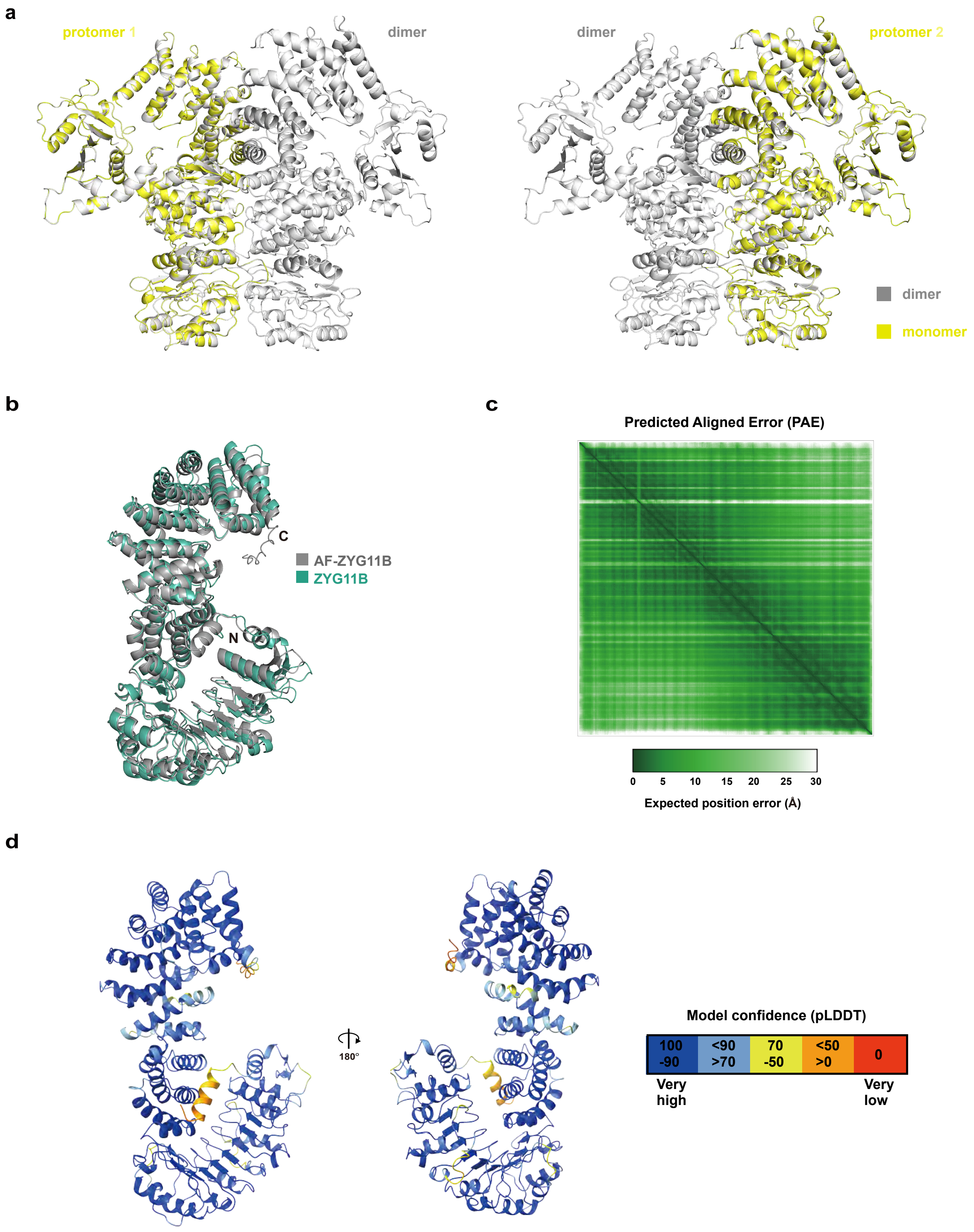

Supplementary Figure 4. Structural alignments of ZYG11B complexes and an AlphaFold model. a, Structural alignment of monomeric ZYG11B-EloB-EloC-substrate (yellow) to protomer 1 and protomer 2 in the dimer (silver), respectively. **b,**Structural alignment of ZYG11B from the monomer (cyan) with the Alphafold-predicted model (AF-Q9C0D3-2-F1;grey). **c,** Predicted aligned error (PAE) plot for the AlphaFold-predicted model of ZYG11B. **d,** AlphaFold-predicted structure of ZYG11B colored by pLDDT confidence scores, shown in two views.

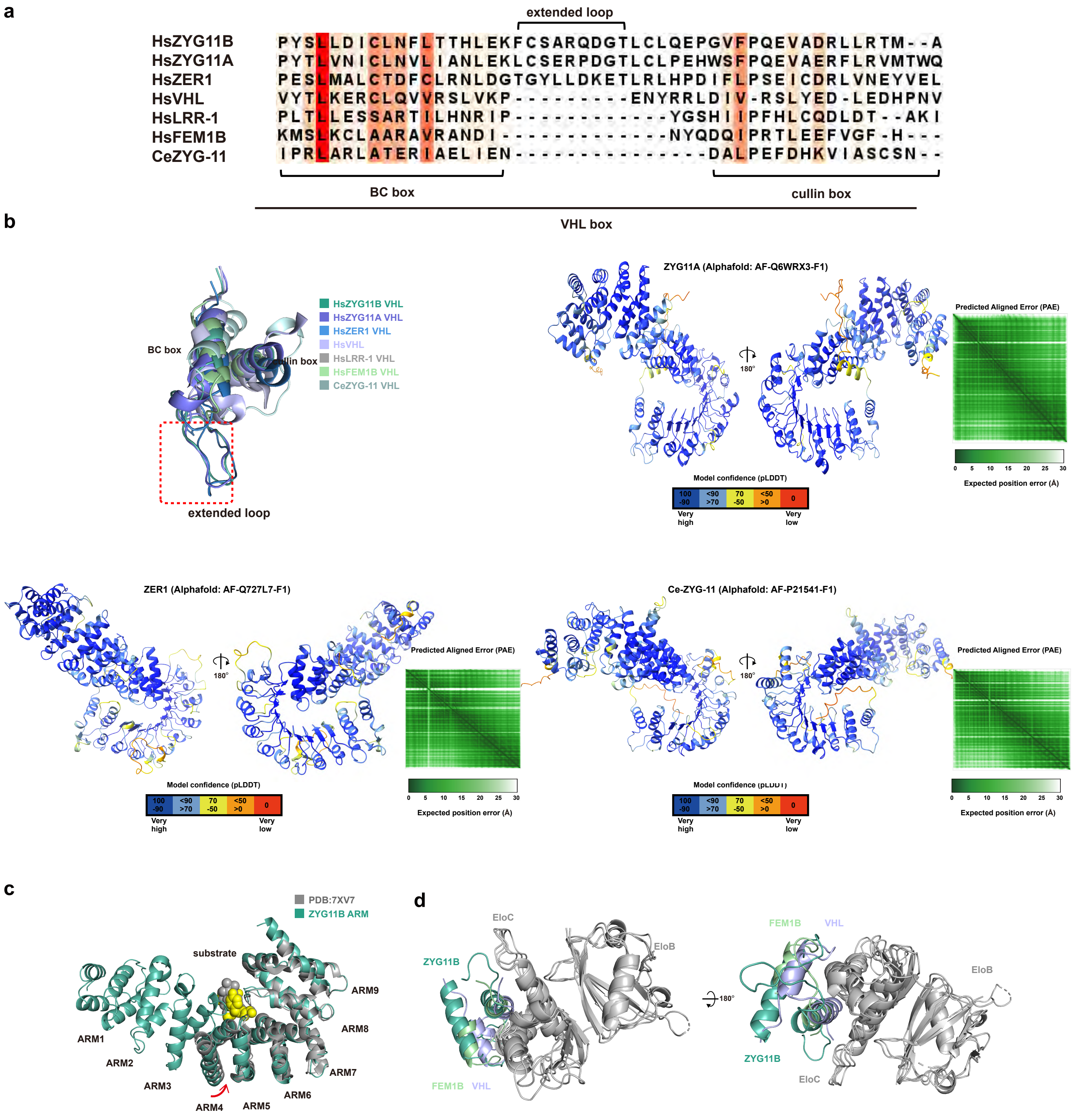

Supplementary Figure 5. Detailed analysis of separated domain of ZYG11B. a, Sequence alignment based on VHL box indicates a longer extended loop connects the BC box and cullin box in members of ZYG11 family except one from Caenor habditis elegans. **b,** Structural superposition of VHL box from various proteins show a similar conformation except the connecting loop in ZYG11B proteins: ZYG11B (this study), ZYG11A (Alphafold: AF-Q6WRX3-F1), ZER1 (Alphafold: AF-Q7Z7L7-F1), VHL (PDB ID: 4WQO), LRR-1 (PDB ID: 7PLO), FEM1B (PDB ID: 8JE2), CeZYG-11 (Alphafold: AF-P21541-F1). Predicted aligned error (PAE) plots and AlphaFold-predicted structures of ZYG11A (AlphaFold: AF-Q6WRX3-F1), ZER1 (AlphaFold: AF-Q7Z7L7-F1) and CeZYG-11 (AlphaFold: AF-P21541-F1), shown in two views and colored by pLDDT confidence scores. **c,** Comparison of the ARM domain structure between this work and previous study (PDB ID: 7XV7). **d,** Structural superposition of different VHL-EloB-EloC complex structures based on EloB-EloC indicates a similar interacting mode in these complexes: ZYG11B (this study), VHL (PDB ID: 4WQO), and FEM1B (PDB ID: 8JE2).

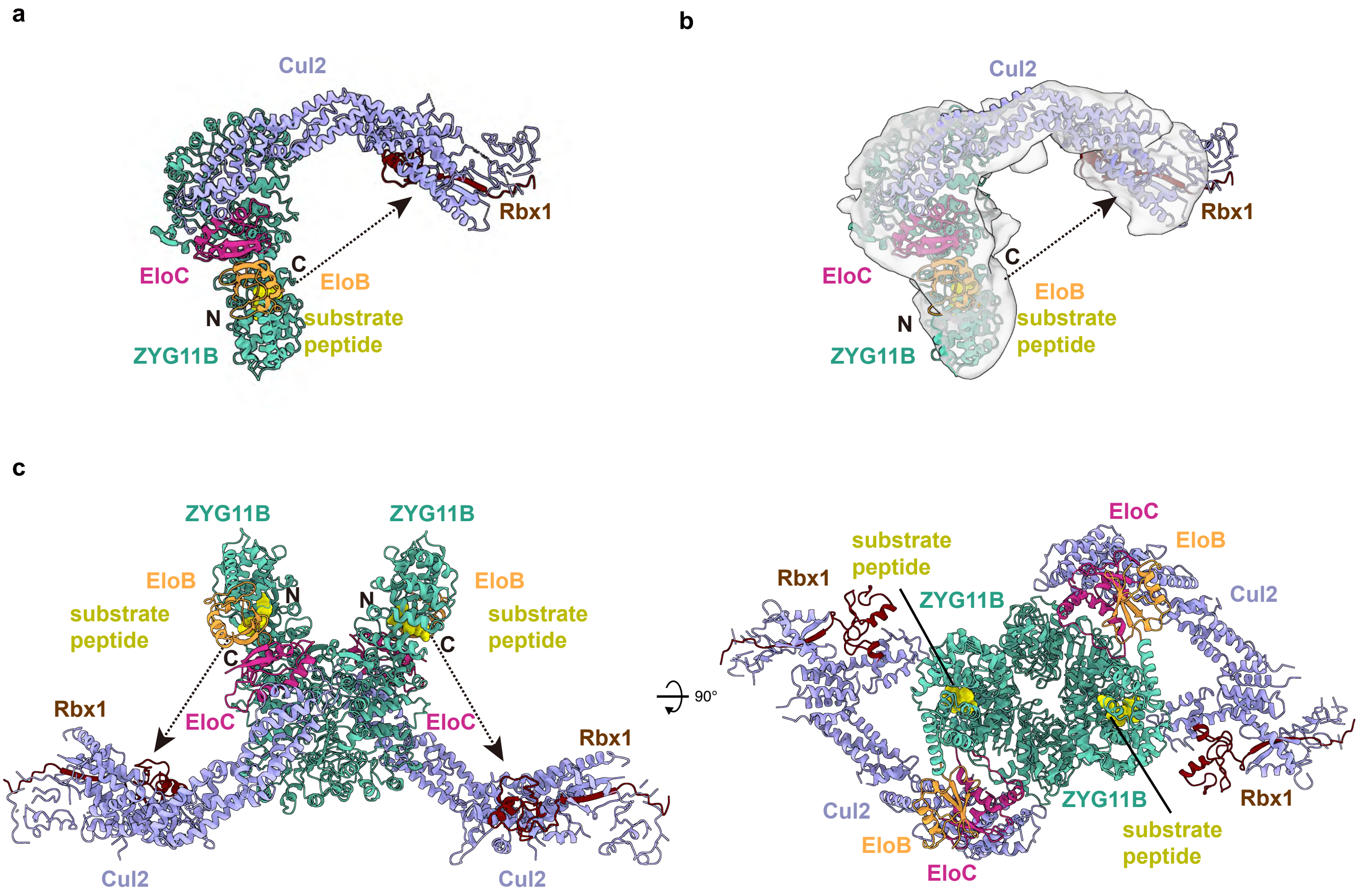

Supplementary Figure 6. 3D models of monomeric and dimeric $CRL2^{ZYG11B}$ with the substrate peptide. a, Docked model of monomeric $CRL2^{ZYG11B}$ based on the VHL-ELoB-EloC-Cul2-Rbx1 structure (PDB: 5N4W). **b,** The monomeric $CRL2^{ZYG11B}$ model fits well into the low-resolution density map of the monomeric complex with substrate peptide. **c,** Two views of the dimeric $CRL2^{ZYG11B}$ model show no steric clashes. The arrow points to the catalytic center of $CRL2^{ZYG11B}$ E3 ligase.

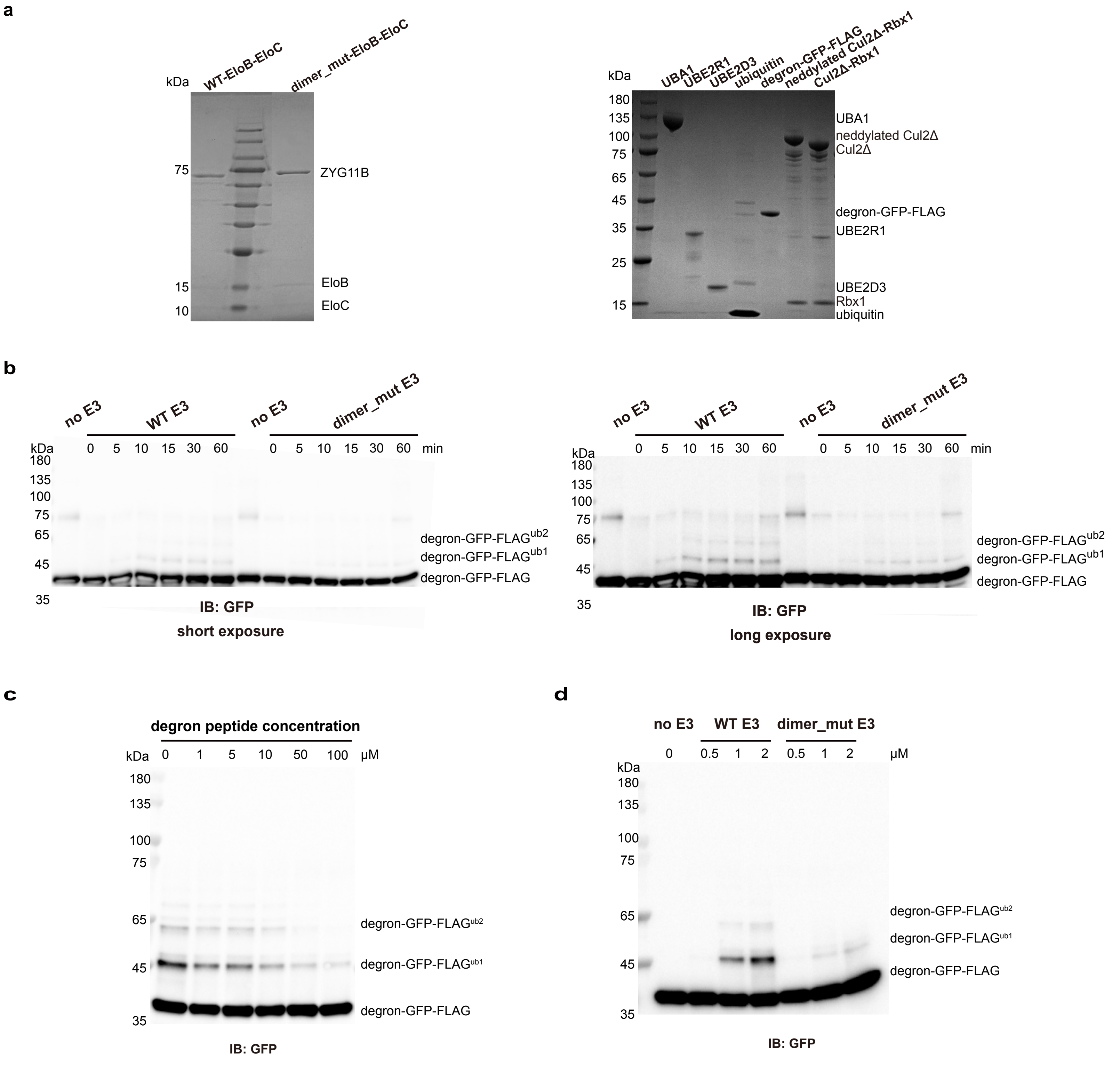

Supplementary Figure 7. The dimer-surface mutation reduces CRL2^{ZYG11B}-mediated mono- and di-ubiquitination of substrates in vitro. a, Coomassie-stained gel showing input proteins for the in vitro CRL2^{ZYG11B} ubiquitination assay. **b,** Time-course ubiquitination of substrate catalyzed by wild-type or dimer-disrupting ZYG11B-EloB-EloC, detected by anti-GFP immunoblotting (left: short exposure, right: long exposure). Wild-type CRL2^{ZYG11B} promoted detectable mono- and di-ubiquiti nation of the substrate within 5 min, whereas the dimer-disrupting mutant showed weaker ubiquitination at all examined time points. **c,** Peptide-competition assay. Increasing concentrations of the competitive peptide (GYFQRGK) reduced substrate (GYFQRGK-GFP) ubiquitination in a dose-dependent manner after 30 min at 37 °C. **d,** CRL2^{ZYG11B} E3 concentration-gradient assay. Increasing E3 levels resulted in progressively enhanced substrate ubiquitination after 30 min at 37 °C, whereas the dimer-disrupting mutant displayed reduced activity at comparable concentrations. Source data are provided as a Source Data file, including all gels and blots.

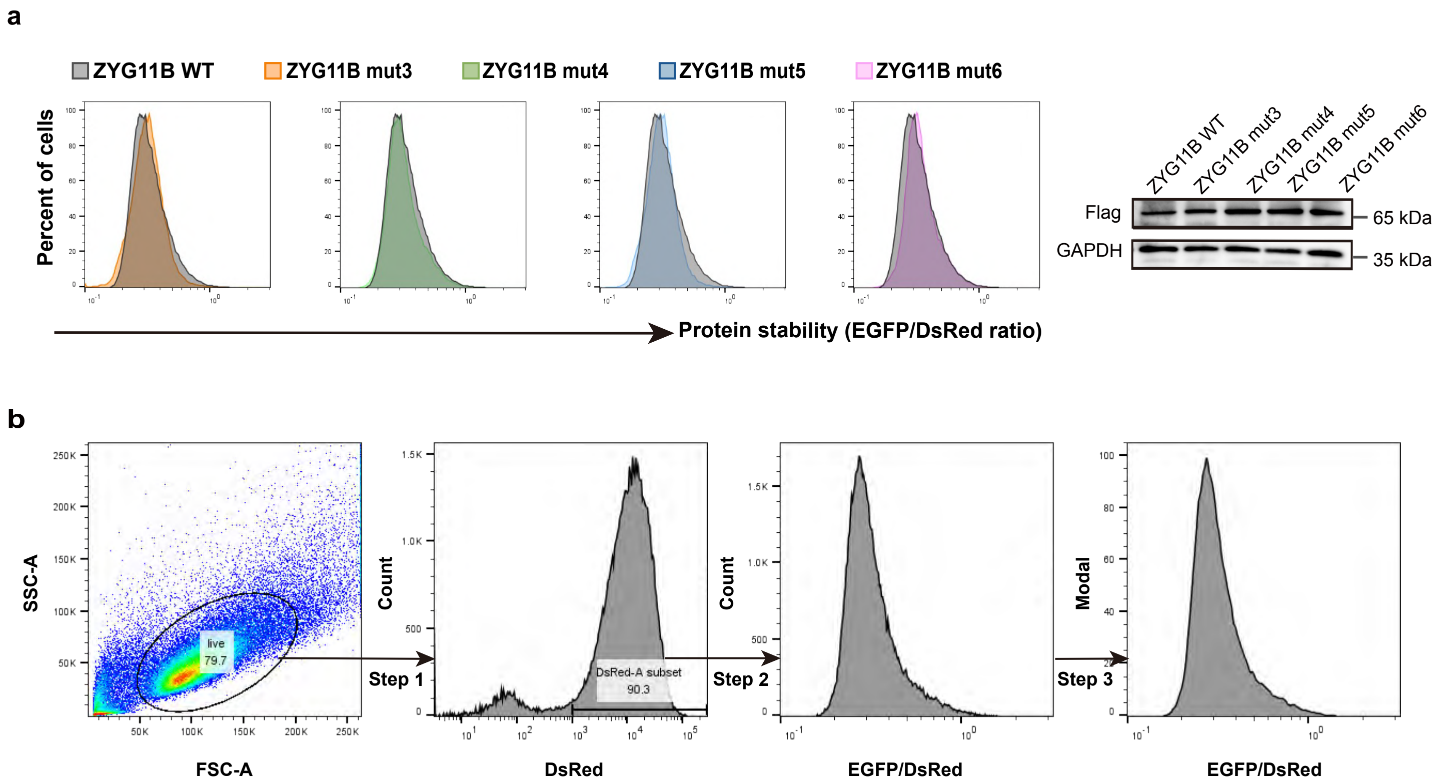

Supplementary Figure 8. Assessment of DBNDD2 degron-EGFP stability upon expression of ZYG11B dimer-interface mutants and FACS garing strategy in GPS assay. a, Stability of DBNDD2 degron-fused EGFP in GPS reporter cells upon exogenous expression of wild-type ZYG11B or dimer-interface mutants (mut3: Q38A/D39A; mut4: D118A; mut5: L150A/D152A/R153A/R342A; mut6: Q249A/F250A). These interface mutations did not noticeably alter reporter stability under the conditions tested. Source data are provided as a Source Data file. **b,** FACS sequential gating/sorting strategies in GPS assay.Cells were first gated on FSC-A and SSC-A to exclude debris and doublets. DsRed-positive cells were then defined as events with DsRed fluorescence intensity > 1×10^3, corresponding to cells carrying lentivirally integrated GPS reporters (Step1). GFP/DsRed fluorescence ratios were subsequently calculated in FlowJo to quantify the stability of the GFP-fused substrate (Step2). In the resulting distributions, the y-axis represents the normalized frequency of cells rather than absolute event counts. Specifically, event counts were normalized such that the total frequency sums to 100% (Step3), thereby reflecting the population-level distribution of relative substrate stability.

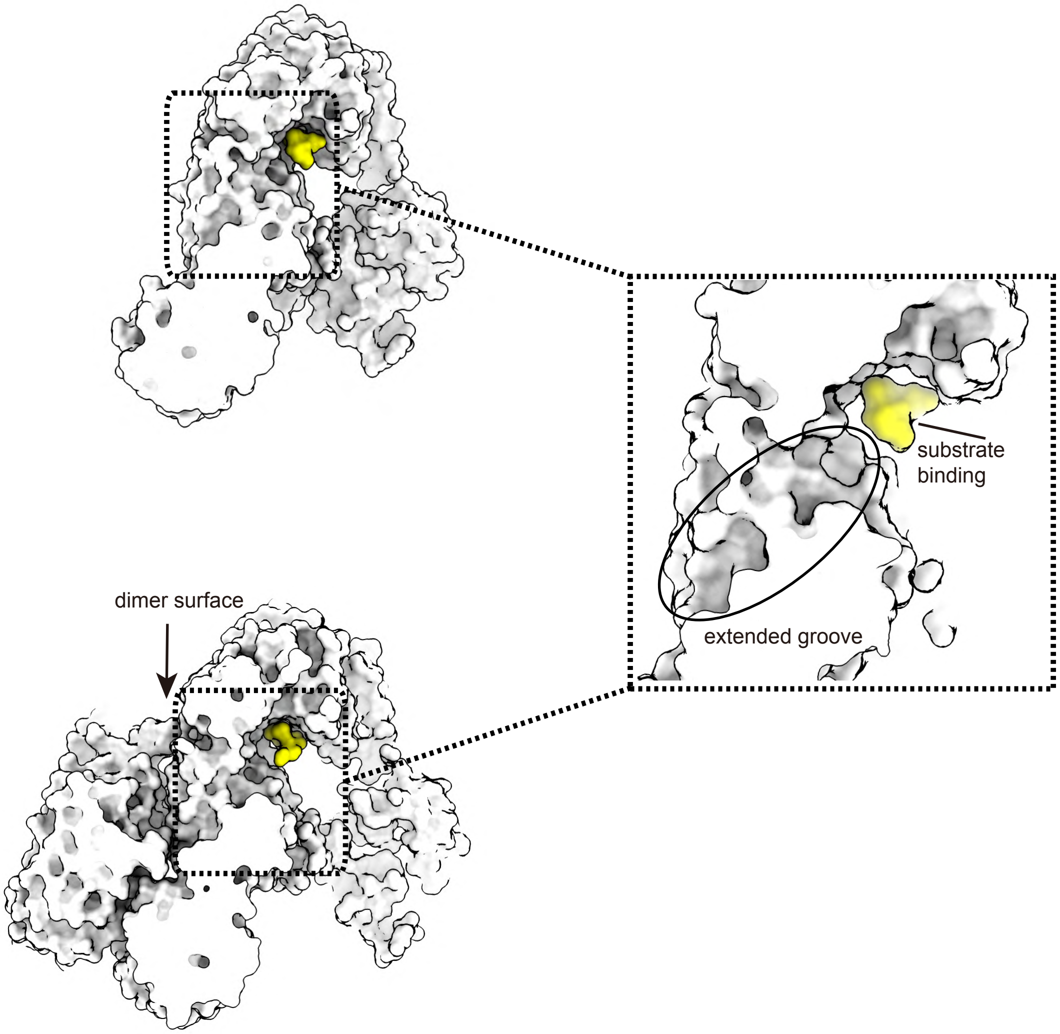

Supplementary Figure 9. The ARM domain groove adjacent to the substrate-binding pocket of ZYG11B. Surface representation of ZYG11B (monomer and dimer, left) showing the substrate-binding pocket and an adjacent groove formed by the ARM domain (outlined) located beneath the pocket and proximal to the dimerization interface. The dashed box indicates an enlarged view of the groove (right), and the substrate peptide is shown in yellow.

Supplementary Table1. Cryo-EM data collection, refinement and validation statistics

	ZYG11B-EloC-EloB-substrate dimer PDB:9LK6 EMDB: EMD-63169	ZYG11B-EloC-EloB-substrate monomer PDB:9LK2 EMDB: EMD-63161
**Data collection processing**		
Magnification	165,000	165,000
Voltage (kV)	200	200
Electron exposure ($e^{-}/Å^2$)	60	60
Defocus rang (μm)	-1.2 ~ -1.8	-1.2 ~ -1.8
Pixel size (Å)	0.8	0.8
Symmetry imposed	C2	C1
Initial particle images (no.)	690,827	690,827
Final particle images (no.)	100,431	99,805
Map resolution (Å)	3.27	3.37
FSC threshold	0.143	0.143
Map resolution range (Å)	-	-
**Refinement**		
Initial model used (PDB code)	None	None
Model resolution (Å)	3.6	3.7
FSC threshold	0.5	0.5
Model resolution range (Å)	3.2-3.6	3.2-3.7
Map sharpening *B* factor (Å)	-104.3	-84.8
Model composition		
Non-hydrogen atoms	14234	7117
Protein residues	1800	900
B factor ($Å^2$)		
Protein	98.47	57.81
R.m.s. deviations		
Bond lengths (Å)	0.003	0.003
Bond angles (°)	0.595	0.615
Validation		
MolProbity score	2.11	2.02
Clashscore	6.86	6.16
Poor rotamers (%)	0	0
Ramachandran plot		
Favored (%)	94.51	95.07
Allowed (%)	5.49	4.93
Disallowed (%)	0	0
