# Supplementary material for: Structures of ZYG11B-EloB-EloC-substrate complex reveal mechanisms of CRL2ZYG11B assembly and function
Source: Nat Commun. 2026 Mar 31;17:4648. doi: 10.1038/s41467-026-71318-x (PMC13201589; doi:10.1038/s41467-026-71318-x)
Supplement: Supplementary file 2 — Reporting Summary [file 41467_2026_71318_MOESM2_ESM.pdf]

nature portfolio

Corresponding author(s): Yong Wang, Pu Gao

Last updated by author(s): Mar 14, 2026

Reporting Summary

Nature Portfolio wishes to improve the reproducibility of the work that we publish. This form provides structure for consistency and transparency in reporting. For further information on Nature Portfolio policies, see our Editorial Policies and the Editorial Policy Checklist.

Statistics

For all statistical analyses, confirm that the following items are present in the figure legend, table legend, main text, or Methods section.

n/a	Confirmed	
☐	☒	The exact sample size (*n*) for each experimental group/condition, given as a discrete number and unit of measurement
☐	☒	A statement on whether measurements were taken from distinct samples or whether the same sample was measured repeatedly
☐	☒	The statistical test(s) used AND whether they are one- or two-sided<br>*Only common tests should be described solely by name; describe more complex techniques in the Methods section.*
☒	☐	A description of all covariates tested
☒	☐	A description of any assumptions or corrections, such as tests of normality and adjustment for multiple comparisons
☐	☒	A full description of the statistical parameters including central tendency (e.g. means) or other basic estimates (e.g. regression coefficient) AND variation (e.g. standard deviation) or associated estimates of uncertainty (e.g. confidence intervals)
☐	☒	For null hypothesis testing, the test statistic (e.g. *F*, *t*, *r*) with confidence intervals, effect sizes, degrees of freedom and *P* value noted<br>*Give P values as exact values whenever suitable.*
☒	☐	For Bayesian analysis, information on the choice of priors and Markov chain Monte Carlo settings
☒	☐	For hierarchical and complex designs, identification of the appropriate level for tests and full reporting of outcomes
☒	☐	Estimates of effect sizes (e.g. Cohen's *d*, Pearson's *r*), indicating how they were calculated

Our web collection on statistics for biologists contains articles on many of the points above.

Software and code

Policy information about availability of computer code

Data collection	SerialEM 3.8
Data analysis	Relion 3.1, Cryosparc v4.7.1, Chimera 1.17.1, ChimeraX 1.6.1, Phenix 1.20.1, Coot 0.9.8.7, Pymol 2.5, Origin 2022, FlowJo 10.8.1.

For manuscripts utilizing custom algorithms or software that are central to the research but not yet described in published literature, software must be made available to editors and reviewers. We strongly encourage code deposition in a community repository (e.g. GitHub). See the Nature Portfolio guidelines for submitting code & software for further information.

Data

Policy information about availability of data

All manuscripts must include a data availability statement. This statement should provide the following information, where applicable:

- Accession codes, unique identifiers, or web links for publicly available datasets
- A description of any restrictions on data availability
- For clinical datasets or third party data, please ensure that the statement adheres to our policy

The data that support this study are available from the corresponding authors upon request. The atomic coordinates and cryo-EM maps have been deposited into the Electron Microscopy Data Bank under accession numbers EMD-63161 (monomer) and EMD-63169 (dimer). The coordinates have been deposited at the Protein Data Bank under accession numbers 9LK2 (monomer) and 9LK6 (dimer).A source Data file is included with this manuscript.

Research involving human participants, their data, or biological material

Policy information about studies with human participants or human data. See also policy information about sex, gender (identity/presentation), and sexual orientation and race, ethnicity and racism.

Reporting on sex and gender	N/A
Reporting on race, ethnicity, or other socially relevant groupings	N/A
Population characteristics	N/A
Recruitment	N/A
Ethics oversight	N/A

Note that full information on the approval of the study protocol must also be provided in the manuscript.

Field-specific reporting

Please select the one below that is the best fit for your research. If you are not sure, read the appropriate sections before making your selection.

- [x] Life sciences
- [ ] Behavioural & social sciences
- [ ] Ecological, evolutionary & environmental sciences

For a reference copy of the document with all sections, see nature.com/documents/nr-reporting-summary-flat.pdf

Life sciences study design

All studies must disclose on these points even when the disclosure is negative.

Sample size	No statistical methods were used to predetermine sample size. The data size for cryoEM was determined by the availability of the microscope time and the particle density on the grids. Sufficient cryoEM data were collected to achieve the reported resolution of map, which is sufficient for model building.
Data exclusions	No data were excluded.
Replication	Each experiment was repeated at least three times in independent experiments. All attempts at replication were successful.
Randomization	No group allocation was needed for functional experiments in this study.
Blinding	Investigators were not blinded to group allocation, because no grouping was needed for this study.

Reporting for specific materials, systems and methods

We require information from authors about some types of materials, experimental systems and methods used in many studies. Here, indicate whether each material, system or method listed is relevant to your study. If you are not sure if a list item applies to your research, read the appropriate section before selecting a response.

Materials & experimental systems

n/a	Involved in the study	
[ ]	[x]	Antibodies
[ ]	[x]	Eukaryotic cell lines
[x]	[ ]	Palaeontology and archaeology
[x]	[ ]	Animals and other organisms
[x]	[ ]	Clinical data
[x]	[ ]	Dual use research of concern
[x]	[ ]	Plants

Methods

n/a	Involved in the study	
[x]	[ ]	ChIP-seq
[ ]	[x]	Flow cytometry
[x]	[ ]	MRI-based neuroimaging

Antibodies

Antibodies used	Anti-GFP antibody (mouse monoclonal, WB 1:2000, ABclonal, Cat# AE012);<br>Anti-Flag antibody (mouse monoclonal, WB 1:2000, ABclonal, Cat# AE005);<br>Anti-GAPDH antibody (mouse monoclonal, WB 1:5000, ABclonal, Cat# AC033);<br>Anti-β-Actin antibody (mouse monoclonal, WB 1:5000, ABclonal, Cat# AC004);<br>HRP-conjugated goat anti-mouse IgG (H+L) secondary antibody (WB 1:10000, ABclonal, Cat# AS003)

Validation	All primary antibodies used in this study are commercially available. According to the manufacturers' datasheets, these antibodies have been validated for Western blot applications in mammalian samples. In our experiments, specific bands corresponding to the expected molecular weights of the target proteins were detected.

Eukaryotic cell lines

Policy information about [cell lines and Sex and Gender in Research]

Cell line source(s)	HEK293-T cells , and sf9 cells were present in the lab at the time this study began.
Authentication	No further authentications were performed for this study.
Mycoplasma contamination	No mycoplasma contamination tests were performed for this study.
Commonly misidentified lines (See ICLAC register)	None.

Plants

Seed stocks	N/A
Novel plant genotypes	N/A
Authentication	N/A

Flow Cytometry

Plots

Confirm that:

- [x] The axis labels state the marker and fluorochrome used (e.g. CD4-FITC).
- [x] The axis scales are clearly visible. Include numbers along axes only for bottom left plot of group (a 'group' is an analysis of identical markers).
- [x] All plots are contour plots with outliers or pseudocolor plots.
- [x] A numerical value for number of cells or percentage (with statistics) is provided.

Methodology

Sample preparation	The cells were harvested, washed with 2%FBS-2mM EDTA-PBS and used for further downstream analysis.
Instrument	Multicolor flow cytometry was performed on the following machines: BD FACS LSRFortessa.
Software	Data was analyzed using FlowJo 10.8.1.
Cell population abundance	Purity was greater than 90% after post sort analysis by flow cytometry.
Gating strategy	Preliminary FSC/SSC gating was used to gate on total population cells. Specific cell population were gate based on the fluorescence signals of EGFP and DsRed.

- [x] Tick this box to confirm that a figure exemplifying the gating strategy is provided in the Supplementary Information.
